# Immune and vascular modulation by HERVs: the role of *CXCR1* and *IL18RAP* in dengue severity progression

**DOI:** 10.3389/fimmu.2025.1557588

**Published:** 2025-03-07

**Authors:** Cristina Santos Ferreira, Alan Tardin Da Silva, Otávio José Bernandes Brustolini, Beatriz Rodrigues Pellegrina Soares, Erika Regina Manuli, Mariana Severo Ramundo, Glaucia Paranhos-Baccala, Ester Cerdeira Sabino, Ana Tereza Ribeiro Vasconcelos

**Affiliations:** ^1^ Laboratório de Bioinformática, Laboratório Nacional de Computação Científica (LNCC/MCTIC), Rio de Janeiro, Brazil; ^2^ Departamento de Moléstias Infecciosas e Parasitárias, Faculdade de Medicina da Universidade de São Paulo, São Paulo, Brazil; ^3^ Instituto de Medicina Tropical, Faculdade de Medicina da Universidade de São Paulo, São Paulo, Brazil; ^4^ Universidade Municipal de São Caetano do Sul, São Caetano do Sul, Brazil; ^5^ Departamento de Clínica Médica, Disciplina de Imunologia Clínica e Alergia, Faculdade de Medicina da Universidade de São Paulo, São Paulo, Brazil; ^6^ Global Medical Affairs Department, bioMérieux SA, Lyon, France; ^7^ Departamento de Patologia, Faculdade de Medicina, Universidade de São Paulo, São Paulo, Brazil

**Keywords:** dengue infection, endogenous retroviruses, severity progression of dengue, vascular leakage, HERV, RNA-seq -RNA sequencing, pro-inflammatory genes, immune modulation

## Abstract

**Introduction:**

Human Endogenous Retroviruses (HERVs), which can be activated by viral infections, have complex roles in gene regulation and immune modulation. However, their contribution to disease progression is not yet fully understood. Dengue fever ranges from mild symptoms to severe cases characterized by plasma leakage and immune dysregulation, providing a relevant context to investigate these interactions.

**Methods:**

This study comes up with a comprehensive analysis of differentially expressed HERVs (DE-HERVs), protein-coding genes (DEGs), and regulatory elements such as microRNAs (DE-miRNA) and non-LTR retroviruses (DE-LINEs and DE-SINEs) derived from the transcriptomes of Brazilian dengue patients across different disease stages.

**Results:**

The results show that DE-HERVs are associated with key genes identified in severe dengue cases, including *ARG1, SLC15A2, COL3A1, SVEP1, CH25H, CST7*, *CXCR1*, *IL18RAP, SORL1*, and *TACR1*, suggesting their role in immune modulation and endothelial permeability. Specifically, the upregulation of *CXCR1* and *IL18RAP* genes in patients who progressed to severe dengue correlates with a complex regulatory network involving down-regulated microRNAs (miRNAs) and non-LTR retroviruses, emphasizing their relevance to inflammation and vascular permeability. MicroRNAs and non-LTR retroviruses were found to regulate these genes differently across dengue stages, with non-LTR elements appearing predominantly in non-severe cases and miRNA expression profiles varying across the comparison groups.

**Discussion:**

These findings improve our understanding of the molecular mechanisms underlying dengue progression and suggest that HERV-related regulatory networks may influence viral infections. Further research is required to clarify the specific roles of HERVs in dengue pathogenesis.

## 1 Introduction

The eukaryotic genome harbors several families of mobile DNA sequences called transposable elements (TE), representing 45% of the genome, these TE being classified as transposons and retroelements (1–6) and can integrate into several genome positions and persist throughout the life (7, 8). The retroelements can be grouped into categories according to the presence or absence of long terminal repeats (LTR), which delimit an internal sequence and contain transcription initiation signals inserted in the genome and multiplied using a ‘copy-and-paste’ mechanism (1, 9, 10). The LTR elements are called endogenous retroviruses (ERVs), constituting one of the largest groups of endogenous viral elements, that originated from the infection of germ cells with ancient retroviruses covering approximately 8% of the human genome (5, 11, 12). Recent studies have explored and demonstrated that human ERVs (HERVs) play a complex role in human health (3, 13), with potential activity in neurological diseases (14–17), cancer (18, 19), inflammatory disease (20–22), and virus infections (23–28).

HERVs have emerged as significant players in the context of viral infections. Studies have shown that Human Immunodeficiency Virus (HIV) infection leads to rapid changes in cellular expression, triggering the activation of endogenous retroviruses such as HERV-K. These changes are associated with immune modulation and viral replication, as HERVs can influence both immune responses and cellular processes (19, 29). HERVs interact with viral proteins like HIV-1 Tat and Rev, affecting the expression of immune-related genes (28). This complex interaction between HIV and HERVs suggests that HERVs might not only contribute to the viral life cycle but also impact the immune system’s ability to control the infection. In the context of influenza A virus (IAV) infection, there is a complex interaction between retroviruses and host immune responses. Upon IAV infection, several HERV elements, including LTR5_Hs, LTR5, LTR12C, and HERV-9NC-int, are significantly up-regulated in A549 infected cells. HERV expression has been shown to modulate immune responses by activating cytokine release through pathways involving NF-κB and TLR2, thereby contributing to both immune activation and the viral response (30). Studies on the role of HERVs in COVID-19 have shown varied patterns of expression, revealing deregulation in specific HERV groups. The HERV-W Env proteins may be expressed on leukocytes in COVID-19 patients, and high expression of HERV-K (HML-2) may stimulate Interferon, correlating both with inflammation and pneumonia severity (31, 32). Furthermore, other studies demonstrated through comparative analyses that some HERV loci were positively regulated by infections such as influenza B, Mayaro, Oropouche, Chikungunya, Zika virus, and dengue serotype 2 (DENV-2) (24, 33). Regarding the association between HERVs and DENV-2, Wang et al. (33) demonstrated that many HERVs and human genes had a significant differential expression in response to DENV-2 infection, possibly being affected by the viral infection (25). Interestingly, the upregulated genes nearby differentially expressed HERVs, were mainly involved in interferon-stimulated antiviral immune responses and may be co-expressed with other genes located near the HERVs (25, 33). These findings establish HERVs as critical modulators of viral pathogenesis, highlighting their potential role in several viral infections, where similar mechanisms may be involved.

Dengue fever (DF) is a rapidly spreading viral disease caused by the Dengue virus (DENV), primarily transmitted by *Aedes aegypti* mosquitoes (34–36). DF is endemic in many tropical and subtropical regions, including Brazil, where seasonal outbreaks occur predominantly during the summer months due to favorable climatic conditions for *Aedes aegypti* mosquito proliferation. In the early months of 2024, Brazil experienced a significant surge in dengue cases, with approximately 6 million probable cases, corresponding to an incidence rate of 2,958.2 per 100,000 inhabitants (37). This represents a 419% increase in probable cases compared to the same period in 2023. Several states, including São Paulo, Minas Gerais, and Paraná, reported public health emergencies due to the overwhelming number of cases and hospitalizations. Recently, the development and approval of a dengue vaccine have emerged as a promising strategy to protect against all four Dengue virus serotypes (38).

The clinical manifestations of dengue range from mild febrile illness to more severe forms such as Dengue Hemorrhagic Fever (DHF) and Dengue Shock Syndrome (DSS), which are characterized by coagulopathy, vascular fragility, thrombocytopenia, and excessive plasma leakage (36, 39, 40). Understanding the factors that contribute to the severity of dengue is important for improving diagnostics, patient management, and therapeutic interventions. In recent years, there has been increasing attention on the genetic and molecular basis of dengue severity, with studies focusing on differentially expressed genes (DEGs) in patients with varying disease outcomes (41–47). These studies have identified key genes involved in immune responses, inflammation, and vascular dysfunction, associated with disease progression (41–43, 48). Identifying these genes is important for distinguishing patients at risk of developing severe dengue, allowing for timely interventions. However, despite these efforts, the precise molecular mechanisms underlying the progression from mild to severe dengue remain not fully understood. This highlights the need for further exploration of additional regulatory factors, such as HERVs, that may modulate host responses to DENV infection and contribute to the severity of the disease. Previous studies have suggested a link between HERV expression and dengue infection, particularly in cellular models (25), but the involvement of HERVs in complex human dengue infection remains poorly understood.

Our research aims to address this gap by examining how HERVs interact with nearby genes, shedding light on their potential role in immune and vascular responses that influence disease severity. We performed genome-wide transcriptome profiling using high-throughput RNA sequencing (RNA-seq) to investigate differentially expressed genes and HERVs associated with dengue progression. The transcriptomic analysis includes data from three clinically distinct groups: patients with mild DENV infection (classical dengue), those with warning signs (WS) who did not progress, and those who developed severe dengue. This analysis enables the identification of key genes involved in immune and vascular pathways, revealing that the synergy between HERVs, microRNAs (miRNAs), and non-LTR retroviruses promotes a complex regulatory network that modulates critical genes such as *CXCR1* and *IL18RAP*. These findings enhance our understanding of the molecular basis of severe dengue, emphasizing the intricate interplay of host genetic regulation in disease severity **Supplementary Figure S1**.

## 2 Materials and methods

### 2.1 Subjects

To explore the molecular mechanisms underlying severe dengue progression, we utilized our previously published transcriptomic datasets (PRJNA1078747) comprising total RNA and small RNA sequencing data from whole blood samples of Brazilian patients. These datasets, provided in FASTQ format, were generated as part of a study aimed at elucidating early mechanisms associated with disease severity in Brazilian patients with dengue infection. The cohort includes 70 individuals, stratified into three clinical groups: (I) classical DENV infection (26 subjects), (II) DENV infection with warning signs (24 subjects), and (III) severe DENV infection (20 subjects). Patients were selected based on specific inclusion criteria, including being over 18 years old, presenting mild symptoms (e.g., fever, headache, myalgia, and rash) for at least seven days, having positive PCR or IgM results, and providing informed consent. Patients who progressed from WS to severe symptoms within 7–14 days were categorized as having severe DENV infection. Total RNA and small RNA libraries were prepared using Illumina library preparation kits and sequenced on the Illumina NextSeq 500/550 platform. Metadata and sample characteristics, are available for consultation at the NCBI BioProject page (https://www.ncbi.nlm.nih.gov/bioproject/1078747). This study were reviewed and approved by the Faculdade de Medicina da Universidade de São Paulo (Approval Number: CAAE 71611417.9.1001.0065 and appreciation number 2.262.437), and the written informed consent for participation was obtained by the participants.

### 2.2 Data processing and identification of differentially expressed elements

The quality of the transcriptome was evaluated using FASTQC (49) and MultiQC (50). Trimming was performed with Trimmomatic (51), and the resulting reads were aligned to the human genome (GRCh38.p13) using STAR (52). For microRNA expression analysis, mature miRNA sequences were mapped from Small RNA sequencing data using Bowtie2 and miRBase v.22 (53–55). For all identified miRNAs, only target genes with experimental validation of miRNA-target binding, according to miRTarBase v.8.0 were recovered (56). Expression analysis of non-LTR retroviruses (long interspersed elements [LINEs], and short interspersed elements [SINEs]) was conducted by mapping sequences from the total RNA library with RepeatMasker v.4.0.5 using Bowtie2 (53, 57). Read quantification was carried out with featureCounts from the Rsubread package (v2.4.3), generating a matrix of read counts for each element (58).

For HERV analysis, reads from the total RNA library were aligned to the human reference genome (GRCH38.p13) using bowtie2 (53), with parameters for sensitive local alignment search (–very-sensitive-local) and up to 100 reported alignments for each read pair (-k 100). The aligned BAM files and the HERV hg38 GTF file were then processed with Telescope (59) to obtain tables with HERV expression values. HERVs with expression below 0.5 counts per million (CPM) were removed.

Differentially expression (DE) analysis of protein-coding genes (DEGs), HERVs (DE-HERV), LINEs (DE-LINE), SINEs (DE-SINE), and microRNAs (DE-miRNA) was performed using the edgeR statistical package (60). The comparison groups included (1) WS over classical (WS-C), (2) Severe over classical (S-C), and (3) Severe over WS (S-WS). The adjusted *p-value ≤*0.05 and Log 2 fold-change ≥ |1.0| were applied as criteria to identify the DE elements for downstream analysis.

### 2.3 Enrichment and interactome analysis

The DEGs near DE-HERVs (Paired) were selected aiming to explore the relationship between the pairs, based on their genomic distance (UpStream and DownStream), considering a median genomic distance of 40 (± 15) kilobases (Kb). For this set of pairs, a functional annotation and pathway analysis were carried out using Gene Ontology (GO), Kyoto Encyclopedia of Genes and Genomes (KEGG), and Reactome databases (61–63). These analyses evaluated pathways potentially related to dengue progression and used Gene Set Enrichment Analysis (GSEA) from the fGSEA package (v1.16.0) (64). Enrichment analysis was conducted using the ClusterProfiler R package v.3.18.0 (65) to generate gene clusters (≥2 genes/cluster). Enriched terms were filtered based on a *p-value* and *q-value* (Benjamini & Hochberg [BH] method) smaller than 0.05. The enriched pathways for biological process (BP), in GO terms, were filtered based on their relationship with immune modulation, cell adhesion, vascular permeability, circulatory system, and ion transport pathways. All of the enriched terms had to be associated with the different stages of DENV infection. The genes present in these pathways were comprehensively analyzed to better understand the progression of dengue fever.

Co-expression modules were also generated using the HumanBase tool (https://hb.flatironinstitute.org/), considering only DEGs paired DE-HERVs, focusing on identifying gene clusters specific to blood tissue networks (66). Genes within each cluster share local network neighborhoods, forming cohesive functional modules with systemic associations. This method uses shared k-nearest-neighbors (SKNN) and the Louvain community detection algorithm to reduce the influence of highly connected genes. Focusing on local network structures emphasizes functional relationships, connecting genes likely to be involved in the same regulatory or biological processes.

### 2.4 miRNA e non-LTR retroviruses expression profile

For the miRNA analysis, only DEGs paired with DE-HERVs involved in immune modulation and vascular permeability pathways were selected to investigate their expression profiles in the severity progression (S-WS group). Selected genes included those with experimentally validated miRNA targets according to miRTarBase or previously described in the literature as miRNA targets. miRNA-gene pairs exhibiting an inverse expression pattern were prioritized. These pairs were examined across all comparisons to assess their expression dynamics throughout dengue severity progression. For the non-LTR retrovirus analysis, only DEGs paired with DE-HERVs in the S-WS group and associated with immune and vascular permeability pathways were selected. The genomic proximity (Kb) between non-LTR retroviruses (LINEs and SINEs) and annotated target genes was considered to evaluate their expression profiles across all comparisons.

## 3 Results

The present study analyzed the transcriptome of 70 Brazilian individuals at different stages of dengue to explore the regulatory mechanisms of HERVs in the immune modulation and vascular permeability related to severe dengue progression. The cohort consists of three dengue stages: 26 patients with classical dengue, 24 patients with Warning signs (WS) of dengue infection, and 20 patients with severe dengue infection. The latter group includes patients initially diagnosed with WS DENV infection who progressed to severe symptoms after 7-14 days. To investigate the differentially expressed HERVs and genes associated with severe dengue progression, we compared the three patient groups as follows: WS over classical (WS-C), Severe over classical (S-C), and Severe over WS (S-WS). The results from the S-WS comparison were particularly emphasized to highlight the progression to severe dengue.

### 3.1 Comprehensive analysis of HERV expression across different dengue patient groups

**Figure 1:**
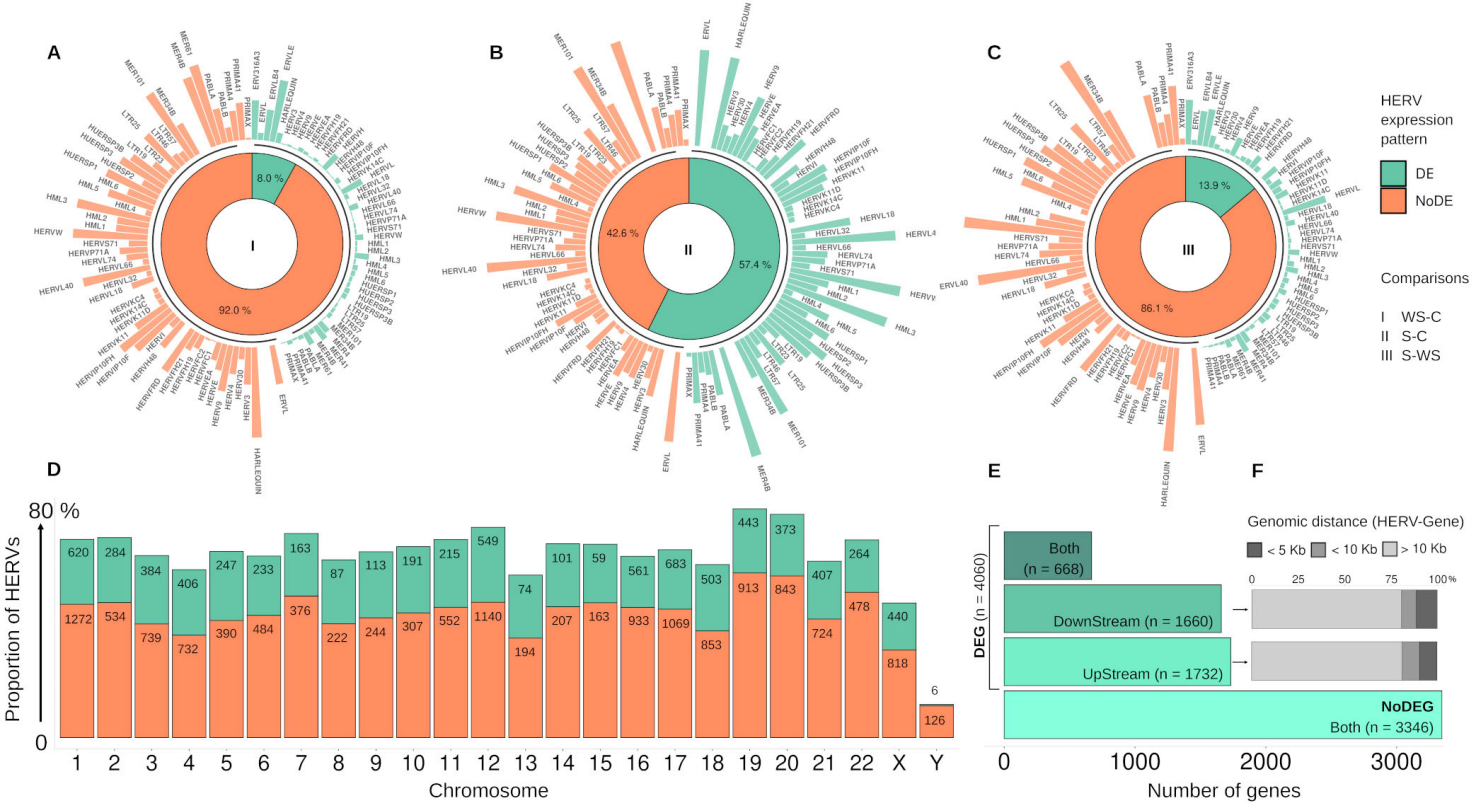
Representative Panel of expressed HERVs distribution. **(A–C)** The plots display the distribution of Differentially expressed (DE) and non-DE HERVs (green and orange, respectively) in the three comparison groups of dengue: (I) WS-C: Warning Signs (WS)-DENV over Classic-DENV; (II) S-C: Severe-DENV over Classic-DENV; and (III) S-WS Severe-DENV over WS-DENV. The innermost layer shows the donut plots with the percentage of DE and non-DE-HERVs, while the subsequent layer displays the distribution of HERV families corresponding to each DE and non-DE-HERVs. **(D)** Plot shows the proportion of DE and non-DE HERV (green and orange, respectively) by chromosome, highlighting the respective number of HERVs. **(E)** Regarding the DE-HERV, plot shows the number of DE-HERV near differentially expressed genes (DEG) or non-DEG, discriminating the number of DEGs by the genomic position of DE-HERV (UpStream, DownStream, or Both). **(F)** Plot represents the percentage of genomic distance categories (less than 5kb; less than 10 KB; and greater than 10 Kb, colored from a dark to light gray) of the DownStream and UpStream DE-HERVs paired with DEGs. The arrow highlights the group of DEG in plot **(E)** used to calculate the distances in plot **(F)**.

Using criteria of Log2FC ≥ |1|, and FDR ≤ 0.05, we identified differential expression (DE) of several HERVs associated with DEGs in all comparison groups (**Supplementary Table S1**). The analysis determined the proportion of expressed HERV across the chromosomes (chr), considering the total number of HERVs provided for the Telescope (59). Except for ChrY, we found the expression of up to 80% of the HERVs by chromosome. Although the distribution of DE and non-DE HERVs followed the same pattern across chromosomes, the number of DE-HERVs on chromosomes 1, 12, and 17 was greater than 1,000 (**Figure 1D**). Regarding the dengue group comparisons, the number of DE-HERVs in the WS-C and S-WS comparisons was less than 15% while the S-C comparison showed a distribution close to 50% of DE and non-DE HERVs (**Figures 1A–C**). The disparity in the S-C group persisted in the family HERVs distribution analysis. While some families (Harlequin, HML3, HERV-W, HERV-L40, MER48, and ERVL) were more represented in the DE-HERV for the S-C group, they were predominant in non-DE HERVs in the other comparison groups. Families that stand out in the DE-HERVs from the WS-C and S-WS comparisons include the more representative families: ERV316A3, ERVLE, HERV-L, and ERVLB4 (**Figures 1A–C**, **Supplementary Table S1**).

In our analysis, the DE-HERVs were distributed across 60 families, with the top five (ERV316A3, HERV-H, ERVLE, HERV-L, and ERVLB4) representing the majority of DE-HERVs identified, accounting for 58% of the total, and displaying distinct distribution patterns across the comparison groups (**Supplementary Table S1**). ERV316A3 was the largest, with 1,064 DE-HERVs, of which 83 were shared across comparisons, and 981 were unique to specific groups: 18 in WS-C, 959 in S-C, and four in S-WS. The HERV-H family included 1,059 DE-HERVs, with 604 shared and 455 unique to the comparisons, including 1 in WS-C, 422 in S-C, and 32 in S-WS. ERVLE comprised 989 DE-HERVs, of which 79 were shared and 910 were exclusive to different groups: 30 in WS-C, 877 in S-C, and 3 in S-WS. The HERV-L family had 699 DE-HERVs, with 81 shared across comparisons and 618 exclusives, including five in WS-C, 612 in S-C, and one in S-WS. Finally, ERVLB4 had 611 DE-HERVs, with 67 shared and 544 unique, distributed as 13 in WS-C, 526 in S-C, and five in S-WS.

**Figure 2:**
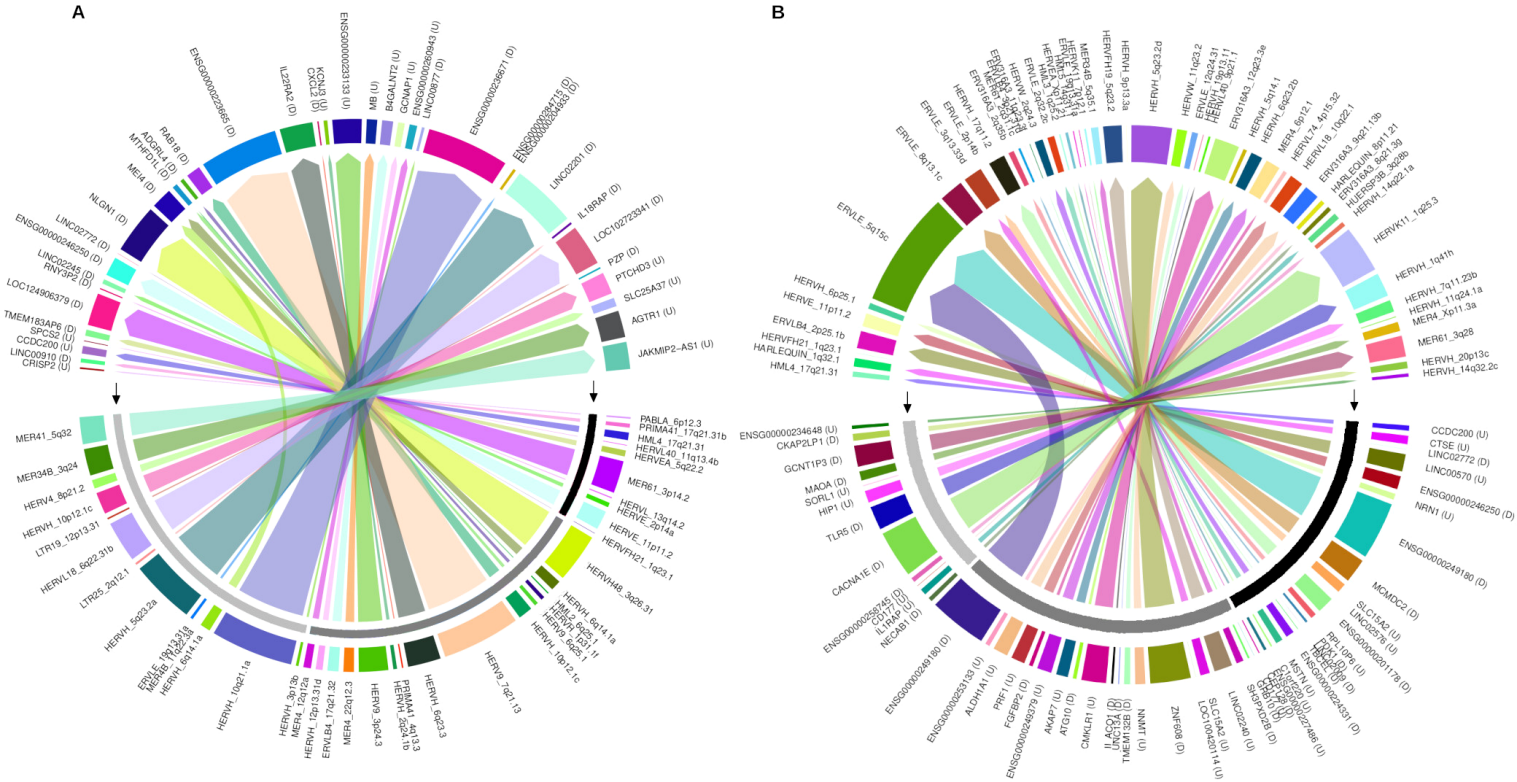
The circus plot represents the paired DE-HERVs and DEGs highlighted in the volcano plot analysis. **(A)** Displays the distribution of highlighted DE-HERVs and their respective paired DEGs. **(B)** Shows the distribution of highlighted DEGs and their respective paired DE-HERVs. The genomic position of DE-HERVs relative to the genes was indicated by letters: D – DownStream and U – UpSream. The outermost colored bar represents each gene or HERV. The middle layer, highlighted by black arrows, indicates the patient comparison groups: a black bar for Warning Signs over Classic, dark gray for Severe over Classic, and light gray for Severe over Warning Signs. The innermost layer illustrates the connections between pairs, with colored arrows originating from the highlighted elements and pointing to their respective pairs.

Highlighting the differentially expressed HERV, a total of 7,406 DE-HERVs (7,381 up-regulated and 25 down-regulated) were identified across the comparisons, of which 54.8% (n= 4,060) were associated with at least one Gene (Paired DE-HERV and DEG) with 99.8% (n=4,052) being up-regulated (**Figures 1A–C, E**). In 18 pairs (0.5%), the expression profiles of DE-HERV and DEG exhibited opposite patterns. Regarding the comparison groups, 4.7% (n= 350) of total DE-HERVs were found in the WS-C comparison, with 22.6% (n= 79) being pairs (Paired DE-HERV and DEG); 82.8% (n= 6,133) in the S-C comparison, with 48.9% (n= 3,001) being pairs; and 12.5% (n= 923) in the S-WS, with 32.4% (n= 299) being pairs (**Supplementary Table S1**), regardless of the comparison we observed that significantly more DE-HERVs were up-regulated than down-regulated. In **Supplementary Figure S2**, we display all DE-HERVs and DEGs, without considering their association, emphasizing only those with lower *p-values* within the pairs (**Supplementary Figure S1**). Furthermore, for these highlighted elements, we show the up-regulated pair HERVH_6q14.1a DownStream to the *MEI4* gene, shared between the S-C and S-WS comparisons (**Figure 2A**); the pair ERVLE_5q15c (up-regulated) DownStream to the *ENSG00000249180* gene (Down-regulated); and the up-regulated pair ERVLE_3q13.33d UpStream to the *SLC15A2* gene, shared between the WS-C and S-C comparisons (**Figure 2B**).

### 3.2 Overview of the genomic proximity and potential regulatory connections between DE-HERVs and nearby genes

The relationship between DE-HERVs and DEG was assessed based on the genomic distance of the pairs. Among the 4,060 paired DE-HERVs and DEGs, 1,732 DE-HERVs were located UpStream of the gene, with a median genomic distance of 40 (± 15) kilobases (Kb). The distribution of these UpStream DE-HERVs was as follows: 9.4% were located within 5 Kb, 9.3% within 10 Kb, and 81.3% beyond 10 Kb. Additionally, 1,660 DE-HERVs were located DownStream, with a median distance of 38 (± 18) Kb, where 11.5% were within 5 Kb, 7.9% within 10 Kb, and 80.6% beyond 10 Kb. Furthermore, 668 DE-HERVs were located both Up and DownStream of the genes (**Figures 1E, F**, **Supplementary Table S1**).

An additional layer of analysis was introduced, considering the common and exclusive paired DE-HERV and DEG across the three different comparisons examined in this study. This analysis identified 31 pairs found simultaneously in S-C and WS-C comparisons, while 222 were shared between S-C and S-WS comparisons (**Supplementary Figure S3**). Considering the DEGs exclusive to each group, we identified 131 pairs unique to the S-WS comparison. These genes were altered in patients with severe DENV infection who progressed from WS disease. In the two analyses involving the classic patient group (WS-C and S-C comparison), there were 3,477 pairs exclusive to the comparison with severe dengue (S-C comparison) and 68 in the comparison with warning signs of dengue that did not progress to severe disease (WS-C).

Furthermore, the accumulation of DE-HERV elements next to specific DEGs was analyzed across the comparisons. As expected the S-C comparison keeps the disparity compared with other groups, presenting the major DE-HERVs next to the same DEG with four DEGs paired with beyond 10 DE-HERVs, 57 DEGs paired with within 9 to 5 DE-HERVs, and 761 DEGs paired with within 4 to 2 DE-HERVs. For WS-C and S-WS were identified up to 3 DE-HERVs were next to the same DEG, as follows: five and one DEGs with three paired DE-HERVs and 12 and 29 DEGs paired with two DE-HERVs in WS-C, and S-WS comparison respectively.

### 3.3 Discerning immune pathways in dengue severity progression

**Figure 3:**
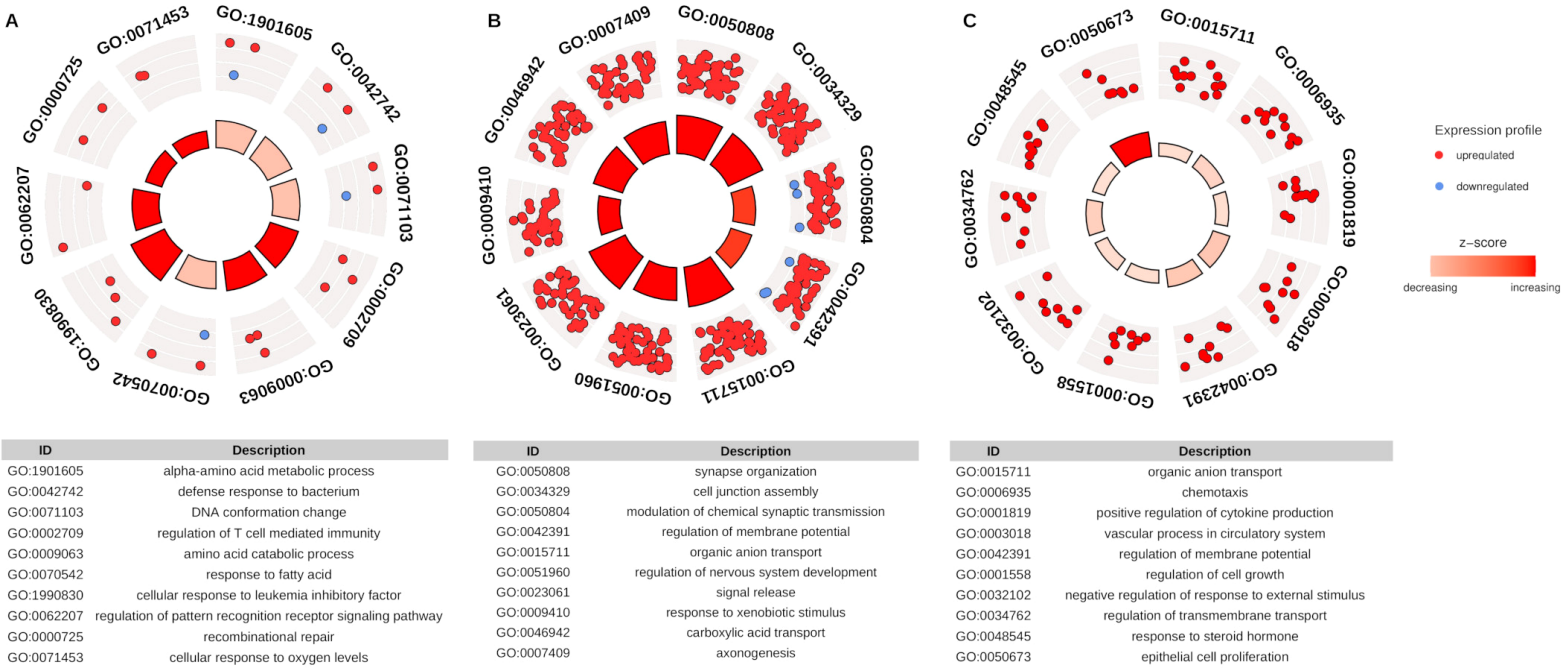
Representative panel of Gene Ontology (GO) enrichment categories for DEGs paired with DE-HERVs. The dots (red - up-regulated and blue - down-regulated) represent the DEG paired with at least one DE-HERV, distributed across the top 10 enriched biological processes pathway in the GO analysis. The inner layer, with a heat bar, indicates the significance of enrichment based on the p-value. **(A)** represents the WS-C group comparison, **(B)** represents the S-C group comparison, and **(C)** represents the S-WS comparison. The categories displayed in the plot are detailed in the respective tables.

Computational enrichment meta-analysis was performed for DEGs paired with DE-HERVs based on GO terms to identify the main biological process (BP) associated with the different stages of DENV infection. A total of 400 GO terms were observed across all comparisons (**Supplementary Table S2**). In the WS-C group, the three major BP categories included alpha-amino acid metabolic process, DNA defense response to bacterium, and conformation change. The S-C comparison presented the highest number of enriched categories and gene counts. The top three BP categories were: synapse organization (65 genes); cell junction assembly (62 genes); and modulation of chemical synaptic transmission (60 genes). For the S-WS comparison, the three main BP categories were organic anion transport (12 genes), chemotaxis (8 genes), and positive regulation of cytokine production (8 genes) (**Figure 3**).

Notably, this result revealed several immune-related pathway enrichments associated with immune modulation, a key feature of the progression of DENV infection severity, highlighting the potential role of HERV regulation in this process. Regarding the biological processes categories enriched to the immune pathways, we identified 22 (5.5%) terms exclusively found in the S-WS comparison group. These enriched terms are associated with 27 DEGs, four of which (*ARG1, CCL24, CXCR1, SLIT2*) were linked to at least seven terms. Additionally, six DEGs (*CH25H, CST7, CXCR1, IL18RAP, SORL1, TACR1*) were exclusively found in the S-WS comparison group.

Intriguingly, the GO term enrichment analysis of DEGs independent of HERV-DEG pairing did not reveal any immune-related pathway (data not shown). To further explore the complexity of gene expression in the immune response without the influence of HERVs an additional KEGG pathway analysis was performed. A total of 18 KEGG pathways were identified across all comparisons (**Supplementary Table S3**). To assess the impact of these pathways on dengue severity progression, 7,909 DEGs from the S-WS group were analyzed, identifying 199 DEGs related to the 16 immune-related pathways. Among these, 40 DEGs were exclusive to S-WS comparison, including two (*CXCR1* and *IL18RAP*) paired with DE-HERVs (**Supplementary Table S4**). The main KEGG pathways identified include the MAPK signaling pathway (49 DEGs), cytokine-cytokine receptor interaction (46 DEGs), and chemokine signaling pathway (24 DEGs).

### 3.4 DE-HERVs modulating genes associated with vascular permeability pathways in dengue severity

**Figure 4:**
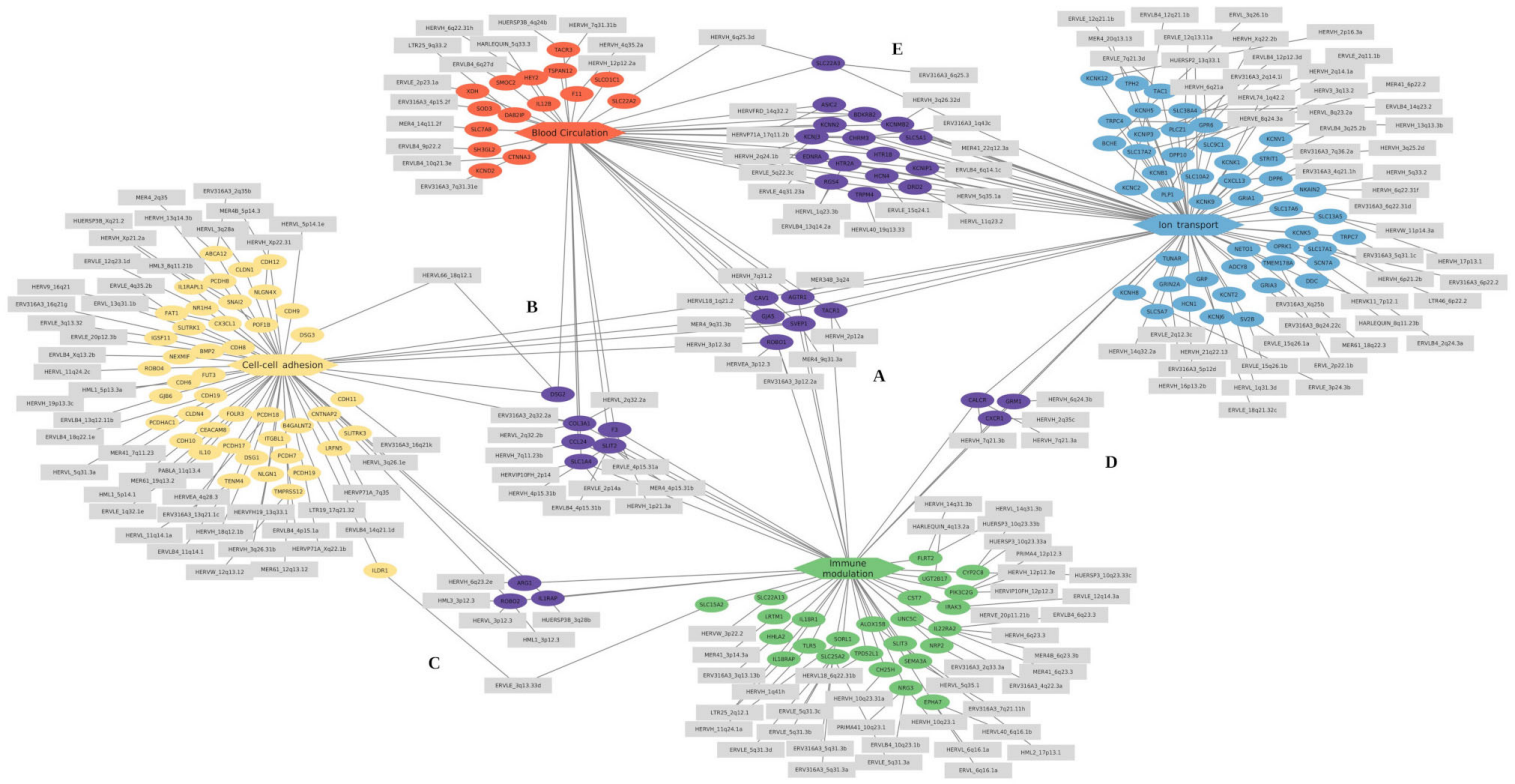
Cnet plots of enriched GO terms of differentially expressed genes related to the immune and vascular modulation categories. The categories are represented as hexagons and positioned at the diagram’s extremities: red represents blood circulation, yellow represents cell-cell adhesion, green represents immune modulation, and blue represents ion transport. The differentially expressed genes (DEGs) exclusive to each category are shown as ellipses in the same color as their respective category. The DE-HERVs are represented as gray rectangles and are connected to their corresponding DEGs. Genes and their associated DE-HERVs shared between categories are highlighted in purple at the central position. The subregions represent shared genes as follows: **(A)** between all four categories, **(B)** between blood circulation, cell-cell adhesion, and immune modulation; **(C)** between cell-cell adhesion and immune modulation; **(D)** between immune modulation and ion transport; and **(E)** between ion transport and blood circulation.

Our analysis further identified DEGs in patients with severe DENV infection within GO biological processes related to blood circulation, cell-cell adhesion, and ion transport. These pathway groups may include DEGs involved in regulating endothelial barrier function, and vascular permeability, typically associated with the severity progression of DENV infection (67, 68). The processes listed in these categories comprise 31 (7.75%) GO terms, of which eight were related to blood circulation, eight to cell-cell adhesion, and 15 from ion transport. Of those, four terms related to blood circulation and three to ion transport are exclusively found in patients who progressed to severe dengue (S-WS group) (**Supplementary Table S2**). The DEGs correlated with each process group as follows: 50 DEGs related to blood circulation, with 21 exclusive of the S-C group, the *TACR1* gene exclusive of the S-WS group, and 28 shared between two groups; 60 associated with cell-cell adhesion, with 46 exclusive of the S-C group and 14 shared; 89 DEGs related to ion transport, with 50 exclusive to the S-C group, three (*CXCR1, GRIA1, TACR1*) exclusive to the S-WS group, and 36 shared between the two comparisons (**Figure 4**).

Regarding the genes shared between the pathway groups, the genes *AGTR1, CAV1, GJA5, ROBO1, SVEP1*, and *TACR1* were common to all groups of enriched GO categories (**Figure 4A**); the genes *CCL24, COL3A1, DSG2, F3, SLC1A4*, and *SLIT2* were shared between three categories (Blood circulation, Cell-cell adhesion, and Immune modulation) (**Figure 4B**); The genes *ARG1, IL1RAP*, and *ROBO2* were shared between cell-cell adhesion and Immune modulation categories (**Figure 4C**); the *CALCR, CXCR1*, and *GRM1* genes were common of Immune modulation and Ion transport categories (**Figure 4D**); and other 16 genes are shared between the Blood circulation and Ion transport categories (**Figure 4E**). The enrichment results obtained from GO analysis were confirmed by the KEGG enrichment, which highlighted the following pathways: Calcium signaling pathway (hsa04020), Viral protein interaction with cytokine and cytokine receptor (hsa04061), and Cytokine-cytokine receptor interaction (hsa04060) (**Supplementary Figure S4**). Additionally, the co-expression modules from HumanBase display pathways related to Inflammatory response and NFkB signaling modules (**Supplementary Figure S5**).

### 3.5 Key genes associated with immune modulation and vascular permeability in dengue severity progression

**Figure 5:**
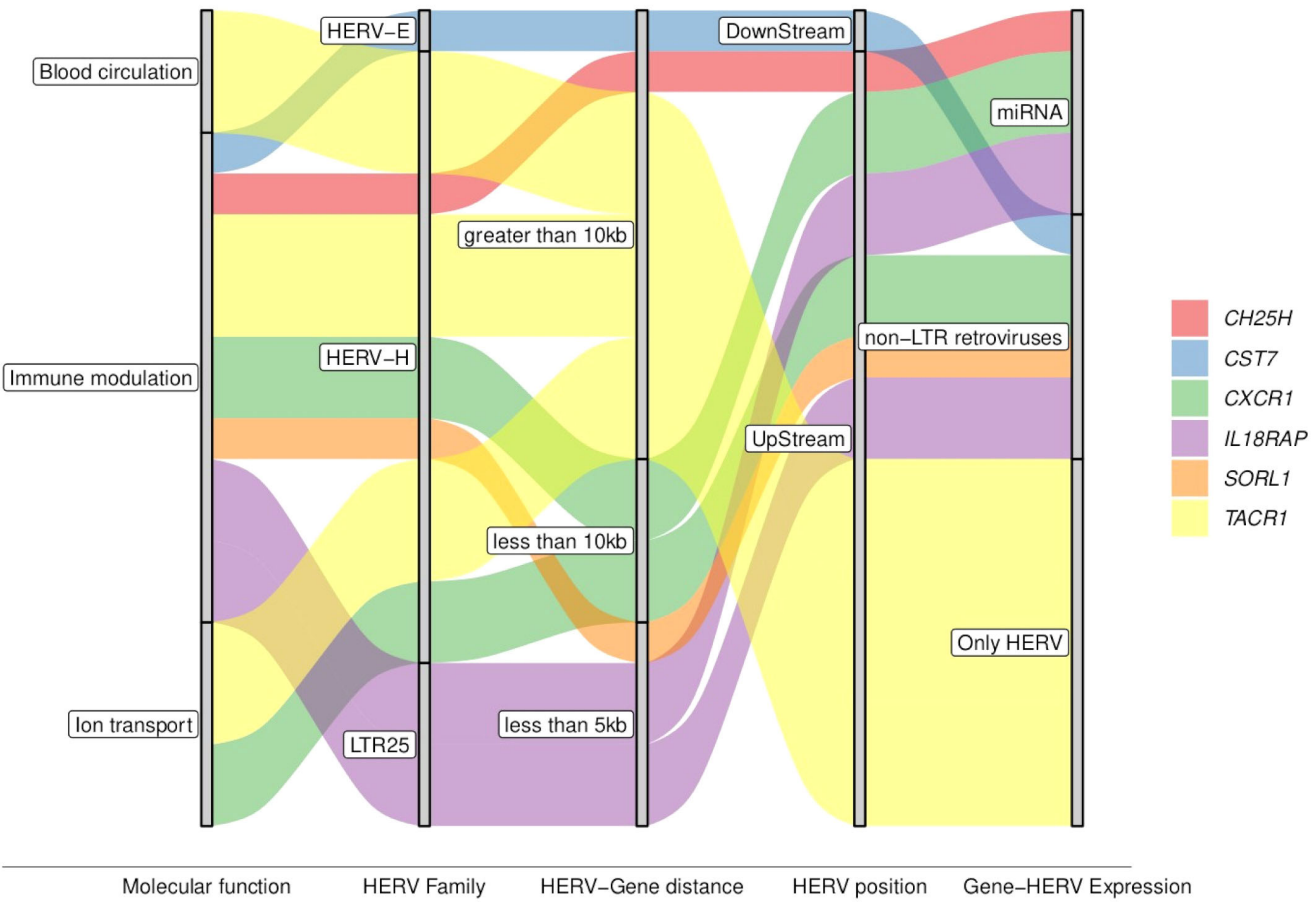
Graphical summary of key attributes of the genes associated with the severe progression of DENV. The colored waves represent the genes distributed across the attributes shown within the gray bars, illustrating shared or specific characteristics.

The modulation of immune response is one of the most common functions regulated by HERV (69, 70). The exclusive evidence of enrichment in the GO immune process in patients who progressed to severe dengue (S-WS group) highlights their role in the dengue severity. Focusing the analysis on the protein-coding DEGs related to the immune process, and exclusive of the S-WS comparison group, we deep investigated the six identified DEGs (*CH25H, CST7, CXCR1, IL18RAP, SORL1, TACR1*) (**Table 1**). Interestingly, the genes *CXCR1*, and *TACR1* were found in more than one pathway group, in addition to immune modulation, including blood circulation and ion transport pathways (**Figure 5**, **Table 1**).

**Table 1:** Description of exclusive genes related to severity progression of dengue.

Gene Symbol	Paired DE-HERV	miRNA	non-LTR retrovirus	HERV Position from Gene	non-LTR Position from Gene	Gene Ontology (GO) Pathways	GO ID
*CH25H*	HERVH_10q23.31a	hsa-miR-26b-5p	–	UpStream	–	chemotaxis	GO:0006935
						cell chemotaxis	GO:0060326
						leukocyte migration	GO:0050900
						leukocyte chemotaxis	GO:0030595
						mononuclear cell migration	GO:0071674
*CST7*	HERVE_20p11.21b	–	20_L1PA7_LINE	DownStream	DownStream	regulation of inflammatory response	GO:0050727
						negative regulation of defense response	GO:0031348
*CXCR1*	HERVH_2q35c	hsa-miR-26b-5p	2_L2b_LINE	UpStream	Inside	chemotaxis	GO:0006935
		hsa-miR-335-5p				positive regulation of cytosolic calcium ion concentration	GO:0007204
		hsa-miR-34a-5p				cell chemotaxis	GO:0060326
						leukocyte migration	GO:0050900
						calcium-mediated signaling	GO:0019722
						leukocyte chemotaxis	GO:0030595
						receptor-mediated endocytosis	GO:0006898
						chemokine-mediated signaling pathway	GO:0070098
						granulocyte chemotaxis	GO:0071621
						myeloid leukocyte migration	GO:0097529
						mononuclear cell migration	GO:0071674
*IL18RAP*	LTR25_2q12.1	hsa-miR-4677-3p	2_MIRb_SINE	UpStream	Inside	regulation of leukocyte mediated immunity	GO:0002703
						regulation of immune effector process	GO:0002697
						positive regulation of cytokine production	GO:0001819
						positive regulation of leukocyte mediated cytotoxicity	GO:0001912
						positive regulation of NF-kappaB transcription factor activity	GO:0051092
						regulation of innate immune response	GO:0045088
						positive regulation of immune effector process	GO:0002699
*SORL1*	HERVH_11q24.1a	–	11_L1MA5_LINE	UpStream	DownStream	positive regulation of cytokine production	GO:0001819
*TACR1*	HERVH_2p12a	–	–	UpStream	–	vascular process in circulatory system	GO:0003018
						regulation of blood circulation	GO:1903522
						positive regulation of cytosolic calcium ion concentration	GO:0007204
						leukocyte migration	GO:0050900
						vasoconstriction	GO:0042310
						blood vessel diameter maintenance	GO:0097746
						regulation of vascular permeability	GO:0043114
						regulation of leukocyte migration	GO:0002685
						regulation of leukocyte proliferation	GO:0070663

The DE-HERVs paired with these six DEGs were distributed across three HERV families: four in the HERV-H family, and one in each of the other HERVs families (HERV-E, and LTR25). Notably, only two DE-HERVs were located within a genomic distance of fewer than 5 kilobases from the genes: LTR25_2q12.1 UpStream of the *IL18RAP* gene and HERVH_11q24.1a UpStream of the *SORL1* gene (**Figure 5**, **Table 1**). Regarding HERV positioning, only one was DownStream of the gene: HERVE_20p11.21b paired with the *CST7* gene (**Figure 5**, **Table 1**).

**Figure 6:**
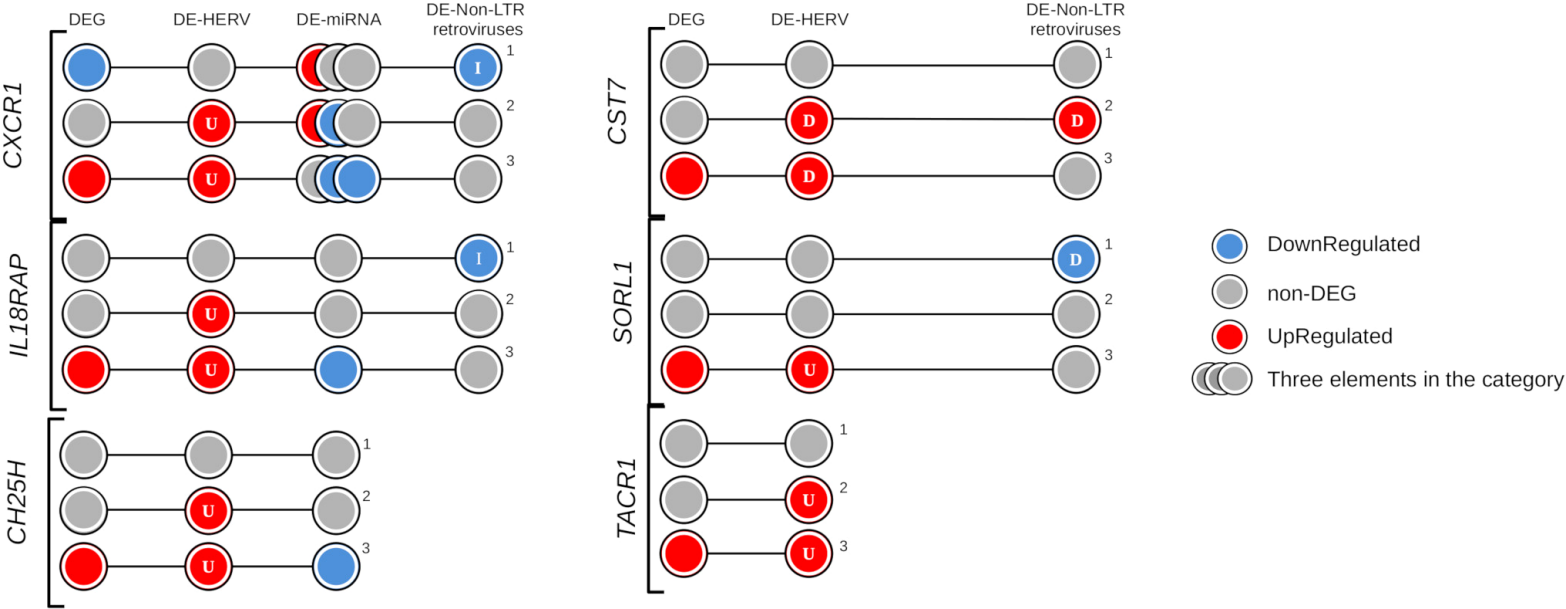
Evidence of regulatory elements in the key genes exclusively identified in the severe progression group of dengue patients. The circles represent differentially expressed (DE) genes and regulatory elements (HERVs, miRNAs, or non-LTR retroviruses) across three comparison groups: (1) Warning Signs over Classic, (2) Severe over Classic, and (3) Severe over Warning Signs. The elements are colored according to their expression profile: blue indicates down-regulation, red indicates up-regulation, and gray represents non-DE profiles. Letters within the circles indicate the position of regulatory elements relative to the gene: I – Inside, D – DownStream, and U – UpStream.

Expanding the analysis to additional regulatory elements near these six genes, we examined the differentially expressed miRNA (DE-miRNA) and non-LTR retroviruses (DE-LINE and DE-SINE), in addition to the DE-HERVs. This comprehensive analysis revealed three genes were possibly regulated by miRNA: *CH25H* (miR-26b-5p)*, CXCR1* (miR-26b-5p, miR-335-5p, and miR-34a-5p), and *IL18RAP* (miR-4677-3p); while four genes were additionally regulated of the non-LTR retroviruses: *CST7* (20_L1PA7_LINE)*, CXCR1* (2_L2b_LINE)*, IL18RAP* (2_MIRb_SINE), and *SORL1* (11_L1MA5_LINE) (**Figure 6**, **Table 1**).

Intriguingly, the genes *CXCR1*, and *IL18RAP* were associated with all three types of regulatory elements investigated, with the *CXCR1* linked to three specific miRNA (miR-26b-5p, miR-335-5p, and miR-34a-5p). For both genes, the identified non-LTR retroviruses exhibited a down-regulation profile in patients from the warning signs group, potentially contributing to their non-DEG status. Furthermore, the down-regulation of miRNAs in the severe progression group appeared to contribute to the up-regulation of *CXCR1* and *IL18RAP* (**Figure 6**). Interestingly, the regulatory mechanisms of *CXCR1* expression are complex and involve not only one HERV but also a LINE element and three miRNAs. In **Figure 6**, the overview of CXCR1 regulation shows the distribution of gene expression profiles and regulatory elements across the comparison groups, helping us understand the differential patterns and potential regulatory layers at each stage of dengue infection. The *CXCR1* gene is reported to have a significant role in inflammation and vascular permeability, which may contribute to disease severity progression (71–73). This is consistent with its up-regulated expression profile and the presence of one DE-HERV (HERVH_2q35c) and two downregulated miRNAs (hsa-miR-26b-5p and hsa-miR-335-5p) in patients who progressed to severe dengue. In contrast, in patients who only exhibit warning signs, *CXCR1* is down-regulated without a corresponding differentially expressed HERV. The gene downregulation is accompanied by a down-regulated LINE element (2_L2b_LINE) at the gene’s location and is associated with the up-regulation of miR-34a-5p, which is linked to decreased expression of CXC motif ligands and receptors, including *CXCR1* (74). These findings underscore the cumulative regulatory effect of HERVs, miRNA, and non-LTR retroviruses on genes involved in immune response and vascular permeability in severe dengue. They provide valuable insights into how the up-regulation of genes related to immune modulation and endothelial dysfunction, modulated by endogenous retroelements, may contribute to the disease’s pathogenesis, emphasizing potential therapeutic targets in severe cases of dengue.

## 4 Discussion

Several attempts have been made to better understand the development of severe dengue through RNA sequencing (RNA-seq) analysis aiming to uncover host gene expression profiles in human peripheral blood (41–47). Numerous studies have demonstrated the activation of human endogenous retroviruses (HERVs) in response to viral infections (24, 26, 27, 75), including arboviruses like dengue and chikungunya (24, 25). The role of HERVs in viral infections remains debated. Some evidence suggests that HERV activation enhances immune responses, offering protection against viral pathogens. However, HERV expression may also exacerbate infection by promoting inflammation and aiding viral evasion of host defenses (76, 77). In this study, we simultaneously analyzed the expression profiles of HERVs and nearby genes from human RNA-seq data to clarify their interconnected roles in dengue infection and disease severity.

A widespread differential expression of HERVs associated with genes was observed across all dengue patient groups, regardless of disease stage (classic, warning signs, or severe). This finding suggests that HERV activation may be influenced primarily by viral infection, independent of the dengue-specific stage. The ability of the dengue virus to trigger the transcription of several HERV loci has been reported (33), additionally, HERVs are known to contain promoter sequences that can drive the expression of nearby genes (13). Therefore, HERVs can regulate gene transcription through several mechanisms, including their Long Terminal Repeats (LTRs), which contain promoter and enhancer sequences, epigenetic modifications, and direct chromatin interactions (11, 78–81). Notably, the HERV associated with *CXCR1* (HERVH_2q35c) overlaps with a Transcription Factor Binding Site predicted as a Regulatory Element from the ORegAnno database (82), suggesting a potential role in transcriptional regulation through transcription factor recruitment or enhancer-promoter interactions. Similarly, the HERV associated with *IL18RAP* (LTR25_2q12.1) overlaps with a CTCF-binding site identified in the ENCODE as a Candidate Cis-Regulatory Elements (83) suggesting the potential contribution of HERVs to the regulation of *IL18RAP* by influencing local chromatin architecture and gene accessibility.

Although these observations align with known HERV regulatory mechanisms the functional significance of this specific HERVs even as the widespread HERV-mediated gene regulation remains unclear, it is increasingly evident that HERVs contribute to key aspects of host immune function (76, 77, 84). The widespread activation of HERVs during dengue infection, coupled with their broad regulatory capacity underscores their potential role as modulators in dengue pathogenesis. One line of evidence supporting the role of HERVs in modulating immune function is the immunosuppressive properties exhibited by envelope proteins from the HERV-H and HERV-K families. These proteins may influence immune response by dampening antiviral activity, potentially creating conditions that benefit viral persistence and replication (85). Intriguingly, five of the key genes found in this study (*ARG1, CH25H, CXCR1, SORL1*, and *TACR1*) were near to differentially expressed genes from the HERV-H family, furthermore, these genes were identified during the severity progression of dengue and have been associated with immune modulation enriched pathways.

The identification of several DEGs such as *ARG1, SLC15A2, COL3A1, SVEP1, CH25H, CST7, CXCR1, IL18RAP, SORL1*, and *TACR1* associated with immune response regulation near HERV loci underscores the significant role of HERV-mediated regulation in dengue pathogenesis and highlight the complex interplay between immune modulation and vascular integrity in the progression of severe dengue. HERV transcription, triggered by viral, infections, has been shown to activate vascular endothelial growth factor receptor (VEGFR1)-dependent pathways, increasing vascular permeability (77, 86). This heightened endothelial permeability facilitates immune activation by promoting transvascular flux, allowing immune cells and mediators to access infected tissues. However, systemic inflammation can exacerbate endothelial damage and vascular permeability, creating a feedback loop where immune modulation and vascular permeability are tightly linked (71, 87–89). Notably, the dengue virus appears to exploit this mechanism, activating HERV expression to enhance vascular permeability and immune activation, potentially tipping the balance to its advantage by promoting viral dissemination and disease severity.

For instance, the *ARG1* gene, which modulates L-arginine metabolism, balances inflammation and tissue repair during viral infections possibly mitigating hyperinflammation or facilitating viral persistence (90–93). In addition, the *SLC15A2* gene is involved in critical immune processes, such as cytokine regulation and antigen presentation, with enriched roles in pathways like vascular transport and defense responses (94, 95). Its interaction with HERV envelope proteins may influence antigen-presenting cell activation and vascular permeability, suggesting dual roles in immune regulation and vascular homeostasis during dengue infection (94, 96). The correlated genes *SLIT2* and *ROBO4* contribute to endothelial stability through the regulation of VE-cadherin expression, a key component of endothelial tight junctions (97, 98). They as part of a potential compensatory mechanism aimed at preserving vascular integrity during infection-induced vascular stress, since the inflammatory stimuli, triggered by an infection, can downregulate the *SLIT2/ROBO4* axis, leading to increased vascular permeability (99, 100). Furthermore, HERVs also influence vascular permeability through genes like *COL3A1, SVEP1*, and *TACR1*, which are linked to endothelial remodeling and plasma leakage. Elevated expression of *COL3A1* has been linked to endothelial dysfunction and vascular remodeling, which may increase vessel fragility and promote plasma leakage in inflammatory conditions (101). Similarly, *SVEP1* potentially modulates vascular permeability in inflammatory contexts, while *TACR1* plays a dual role in immune and vascular contexts by promoting the proliferation and survival of lymphoid precursors and enhancing B-cell activation via MAPK pathway co-stimulation, important for immune differentiation (102). Additionally, *TACR1* signaling supports endothelial proliferation, nitric oxide production, and *VEGF* expression, contributing to angiogenesis and increased vascular permeability (103). The *CXCR1* and *IL18RAP* play significant roles in mediating immune responses and vascular permeability. These genes, regulated by associated DE-HERVs, drive inflammatory cytokine production and chemotaxis, contributing to the immune and vascular dysregulation observed in severe dengue.

The *CXCR1* gene encodes a receptor for IL-8 and plays an important role in mediating immune responses. IL-8 enhances vascular permeability and acts as a strong chemoattractant for neutrophils and cytotoxic memory CD8+ T cells, both of which contribute to endothelial dysfunction and plasma leakage in dengue hemorrhagic fever (DHF) (71–73). Along with IL-8, IL-18 can inhibit the antiviral effects of interferon-α, potentially promoting viral dissemination and worsening disease severity (104). Moreover, IL18RAP, which strengthens IL-18 signaling, plays a complementary role by promoting the production of pro-inflammatory cytokines and driving immune activation (71). In DENV-2 infection, these chemokines are upregulated, stimulating monocyte adherence and promoting angiogenesis through interaction with CXCR1 and CXCR2. This dynamic interplay emphasizes the role of *CXCR1* in amplifying immune and inflammatory responses, contributing to vascular permeability during dengue infection (72). The up-regulation of the *CXCR1* gene, along with the *IL18RAP* gene, may mediate the effects of IL-8, IL-18, IL-1β, and other chemokines by reducing the activation and effector functions of T cells, while also acting as a key chemotactic signal for NK cells (105–107). Reduced *CXCR1* gene expression correlates with decreased NK cell counts and impaired viral control, as observed in early HIV infection (44). The up-regulation of *CXCR1*, and *IL18RAP* in the severe progression of dengue, coupled with associated DE-HERVs, and an additional regulatory layer with miRNA and non-LTR retroviruses suggests a complex role of these genes in intensified inflammatory response that may further exacerbate vascular leakage and immune-mediated tissue damage, underlining their roles in immune modulation, possibly contributing to the severe progression of dengue. These findings highlight the role of cumulative regulation emphasizing their potential involvement in immune modulation and their association with dengue severity progression.

Regarding the potential for confounding in the complex immunological modulation of gene expression during viral infections, we carefully assessed possible confounding factors that could influence the observed differential expression of *CXCR1* and *IL18RAP*. To this end, we investigated the gene expression in the context of dengue severity progression independently of HERV-DEG pairing and performed pathway-level analysis to account for broader immune activation effects. Notably, the GO term enrichment analysis of DEGs not paired with HERVs did not identify any immune-related pathways, reinforcing the specificity of HERV-associated modulation. Additionally, 16 different KEGG pathways were identified suggesting that other host or viral factors could contribute to immune modulation. Furthermore, KEGG analysis revealed that *CXCR1* and *IL18RAP* are associated with immune-related pathways, consistent with the GO enrichment analysis of HERV-DEGs, particularly in cytokine-cytokine receptor interactions and chemokine signaling. Moreover, despite possible confounding factors, the association of *CXCR1* and *IL18RAP* with differentially expressed HERVs, as previously reported (108, 109), highlights the potential role of HERVs in fine-tuning immune responses during viral infection, warranting further investigation into their contribution to disease progression.

While our study primarily focuses on transcriptomic data, *in vivo* evidence from other disease models supports the modulation of *CXCR1* and *IL18RAP* genes during viral infections. Studies on COVID-19 have shown a correlation between HERV-W ENV expression and pro-inflammatory cytokine levels, reinforcing the role of HERVs in immune activation and disease severity (108, 110). Even with the challenges of directly interpreting *in vitro* transcriptomic findings in a physiological context, the concordance of our results with prior *in vivo* studies strengthens the suggestive potential role of *CXCR1* and *IL18RAP* upregulation in severe dengue. Given the parallels between HERV activation and immune responses in other viral infections, such as COVID-19 and HIV, therapeutic strategies targeting the HERV-CXCR1-IL18RAP axis could be promising. For instance, CXCR1 inhibitors and IL-17 blockers, previously explored in COVID-19, offer valuable insights into potential interventions for modulating immune responses (109). Moreover, therapies directly targeting HERVs, such as temelimab (a monoclonal antibody neutralizing HERV-W ENV) and antiretroviral drugs like abacavir and zidovudine, have demonstrated efficacy in reducing inflammation and suppressing HERV replication in other disease contexts (111–113).

It is important to emphasize that this study is exploratory, relying solely on transcriptomic data, without experimental validation. As such, our findings should be interpreted as hypothesis-generating rather than definitive. The associations between HERV expression, *CXCR1*, *IL18RAP*, and dengue severity represent insights that should be further investigated through functional assays and patient-derived immune cell models. Further investigation is required to understand the mechanistic role of HERV-associated immune modulation and assess whether targeting the HERV-CXCR1-IL18RAP axis could offer novel therapeutic strategies for severe dengue.

## 5 Conclusion

This study presents compelling evidence that HERVs, miRNAs, and non-LTR retroviruses contribute to the regulatory mechanisms of gene expression in dengue, particularly in severe disease progression. Key findings demonstrate the up-regulation of immune-related genes, such as *CXCR1* and *IL18RAP*, along with HERV elements and down-regulated miRNAs in severe dengue patients. In contrast, non-severe cases exhibited down-regulation or lack of differential expression of these genes near non-LTR retroviruses, which may function as suppressors. These findings underscore the dual role of these elements in either exacerbating or mitigating disease progression, highlighting the importance of endogenous retroelements in host-pathogen interactions. This intricate interplay presents significant challenges, such as disentangling the specific contributions of each regulatory element and understanding whether these interactions primarily benefit host defense or facilitate viral replication. Overall, this work enhances our understanding of the genomic and regulatory dynamics underlying dengue pathogenesis.

## Data Availability

Publicly available datasets were analyzed in this study. This data can be found here: SRA-NCBI under the accession number PRJNA1078747. Access link: https://www.ncbi.nlm.nih.gov/bioproject/1078747.

## Supplementary material

Supplementary Figure 1 Schematic Overview of HERV-Associated Regulatory Networks in Dengue Progression. **(A)** Representation of the cohort, illustrating the number of patients and how they were categorized into comparison groups (WS-C, S-C, and S-WS). **(B)** An overview of the key molecular elements analyzed for differential expression, including Human Endogenous Retroviruses (HERVs), protein-coding genes (DEGs), microRNAs (DE-miRNAs), and non-LTR retroviruses (DE-LINEs and DE-SINEs). **(C)** A summary of the main findings related to four studied pathways, highlighting the regulatory network involving *CXCR1* and *IL18RAP*, which are linked to immune modulation and vascular permeability in severe dengue cases.

Supplementary Figure 2 Volcan plot of differentially expressed elements (HERV and Gene) across the dengue comparison groups. The plot demonstrates the dispersion of the HERVs **(A-C)** and genes **(D-F)** by comparing the log2FC and significance (−log10(p-value)). Red dots indicate differentially expressed elements that were up-regulated with log2FC ≥ 1 and adjusted p-value ≤ 0.05. Blue dots indicate elements that were down-regulated with log2FC ≤ -1 and adjusted p-value ≤ 0.05. The highlighted named elements represent the top ten elements with the lowest p-values within the paired associations. In panels **(D-F)**, the genomic position of DE-HERVs relative to the nearby genes is indicated by letters: D – DownStream and U – UpSream.

Supplementary Figure 3 Venn diagram illustrating the relationship between paired DE-HERVs and DEGs in dengue studies. The Venn diagram displays the relationship among the three comparisons analyzed. Each section of the diagram indicates the number and percentage of pairs shared or unique in each comparison group. WS_C: Warning Signs (WS)-DENV over Classic-DENV; S_C: Severe-DENV over Classic-DENV; and S_WS Severe-DENV over WS-DENV.

Supplementary Figure 4 Enriched KEGG pathways of differentially expressed genes paired with DE-HERV. Distribution of enriched pathways of the genes with a similarity group of 0.8. The heatmap color indicates the lowest false discovery rate (FDR) and the circle sizes correspond to gene count. Generated by ^©^ STRING Consortium 2024 (https://string-db.org/).

Supplementary Figure 5 Co-expression module from blood tissue-specific network-based functional interpretation of differentially expressed genes paired with DE-HERV. The network represents genes, with the circle size indicating their connection count. The network colors denote the co-expression modules (M). The table details the information corresponding to each module. Generated by the HumanBase tool (https://hb.flatironinstitute.org/) (66).
