# Supplementary figures and images for: Immune and vascular modulation by HERVs: the role of CXCR1 and IL18RAP in dengue severity progression
Source: Front Immunol. 2025 Mar 7;16:1557588. doi: 10.3389/fimmu.2025.1557588 (PMC11925782; doi:10.3389/fimmu.2025.1557588)

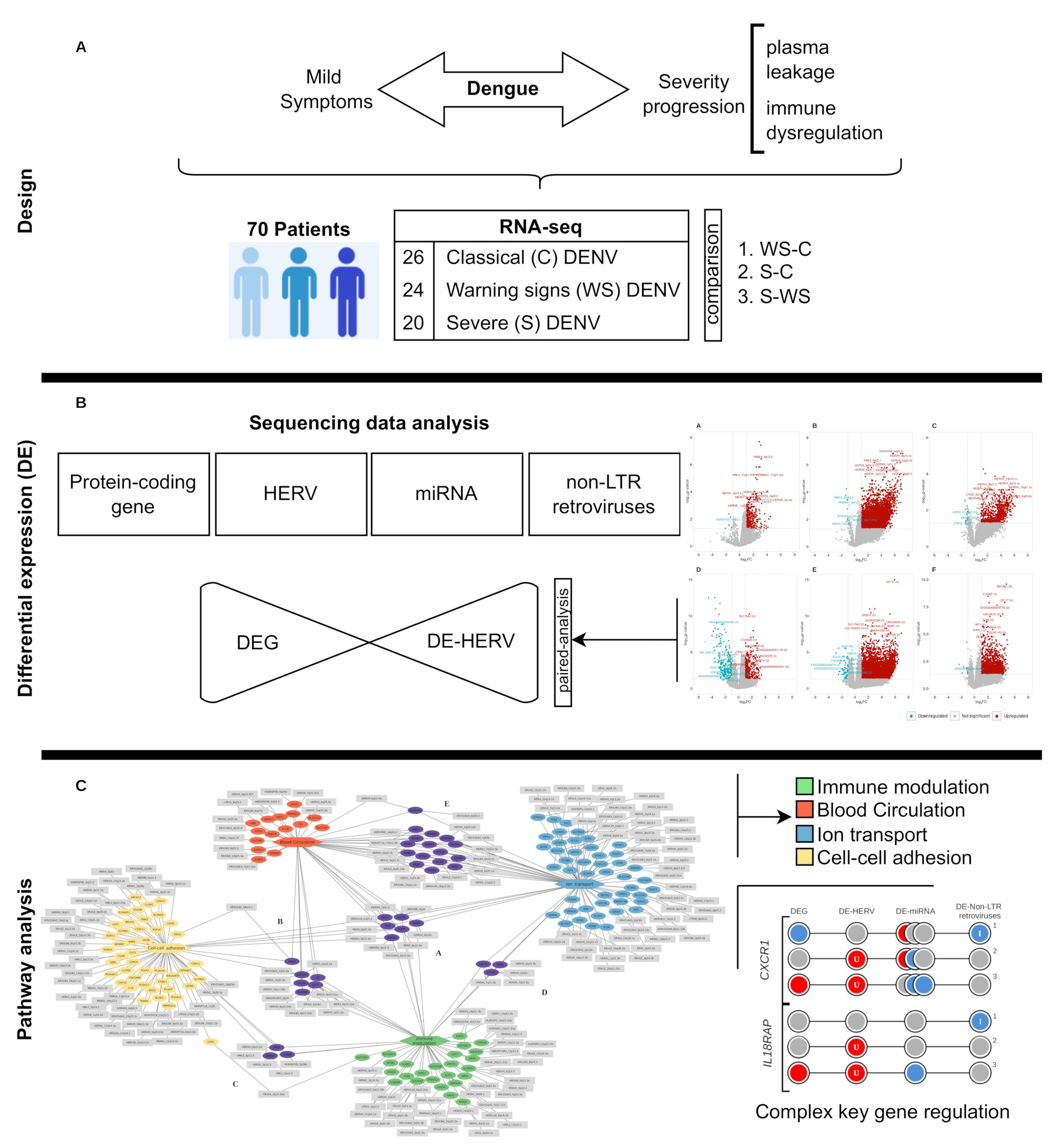

Supplement: Supplementary Figure 1 — Schematic Overview of HERV-Associated Regulatory Networks in Dengue Progression. (A) Representation of the cohort, illustrating the number of patients and how they were categorized into comparison groups (WS-C, S-C, and S-WS). (B) An overview of the key molecular elements analyzed for differential expression, including Human Endogenous Retroviruses (HERVs), protein-coding genes (DEGs), microRNAs (DE-miRNAs), and non-LTR retroviruses (DE-LINEs and DE-SINEs). (C) A summary of the main findings related to four studied pathways, highlighting the regulatory network involving CXCR1 and IL18RAP, which are linked to immune modulation and vascular permeability in severe dengue cases. [file Image1.jpg]

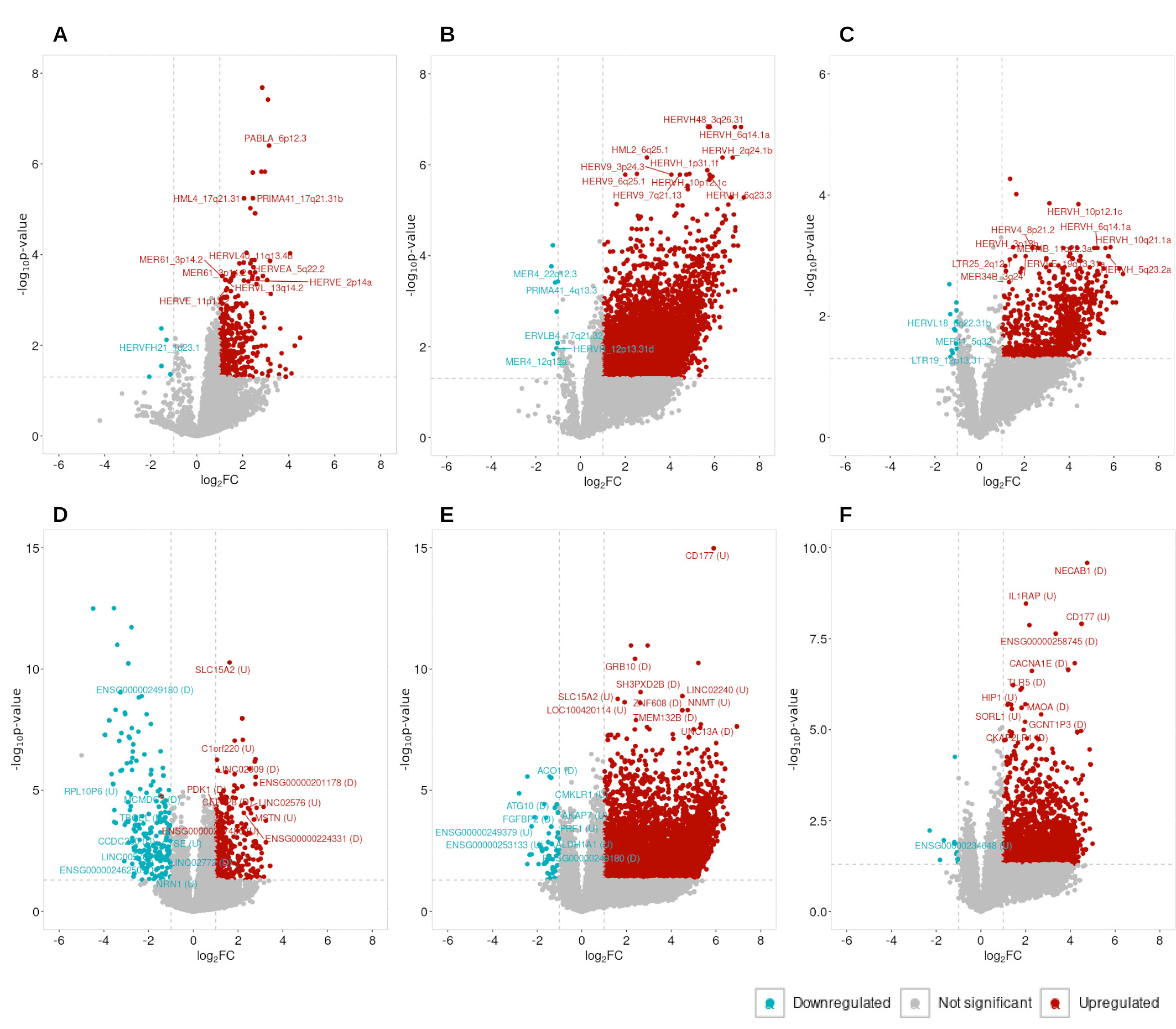

Supplement: Supplementary Figure 2 — Volcan plot of differentially expressed elements (HERV and Gene) across the dengue comparison groups. The plot demonstrates the dispersion of the HERVs (A-C) and genes (D-F) by comparing the log2FC and significance (−log10(p-value)). Red dots indicate differentially expressed elements that were up-regulated with log2FC ≥ 1 and adjusted p-value ≤ 0.05. Blue dots indicate elements that were down-regulated with log2FC ≤ -1 and adjusted p-value ≤ 0.05. The highlighted named elements represent the top ten elements with the lowest p-values within the paired associations. In panels (D-F), the genomic position of DE-HERVs relative to the nearby genes is indicated by letters: D – DownStream and U – UpSream. [file Image2.jpg]

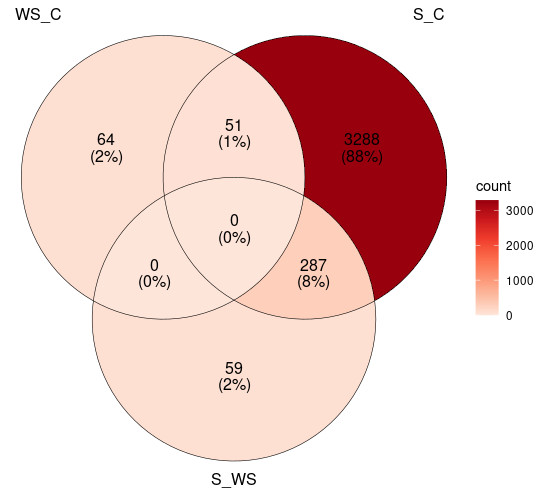

Supplement: Supplementary Figure 3 — Venn diagram illustrating the relationship between paired DE-HERVs and DEGs in dengue studies. The Venn diagram displays the relationship among the three comparisons analyzed. Each section of the diagram indicates the number and percentage of pairs shared or unique in each comparison group. WS_C: Warning Signs (WS)-DENV over Classic-DENV; S_C: Severe-DENV over Classic-DENV; and S_WS Severe-DENV over WS-DENV. [file Image3.jpg]

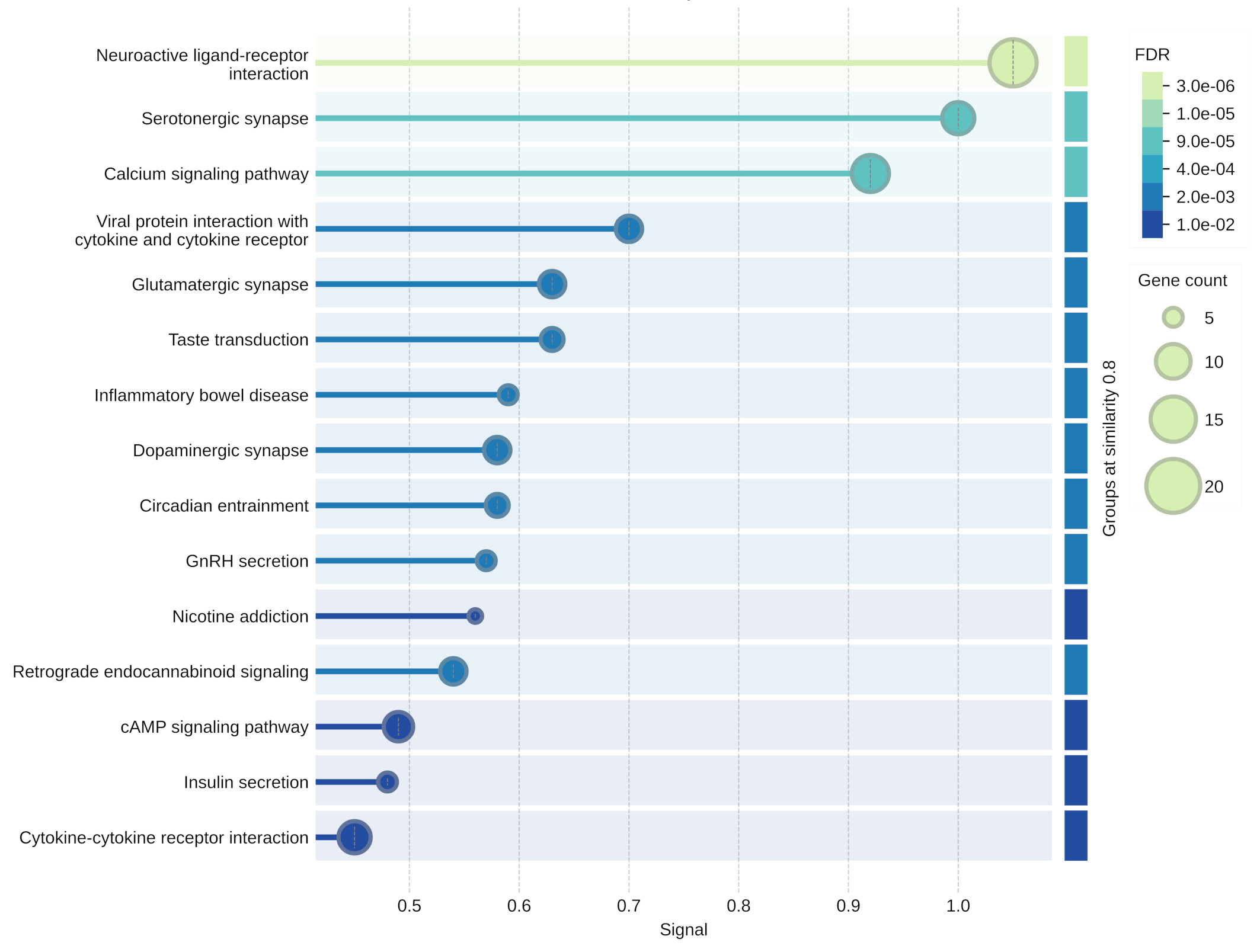

Supplement: Supplementary Figure 4 — Enriched KEGG pathways of differentially expressed genes paired with DE-HERV. Distribution of enriched pathways of the genes with a similarity group of 0.8. The heatmap color indicates the lowest false discovery rate (FDR) and the circle sizes correspond to gene count. Generated by © STRING Consortium 2024 (https://string-db.org/). [file Image4.jpg]

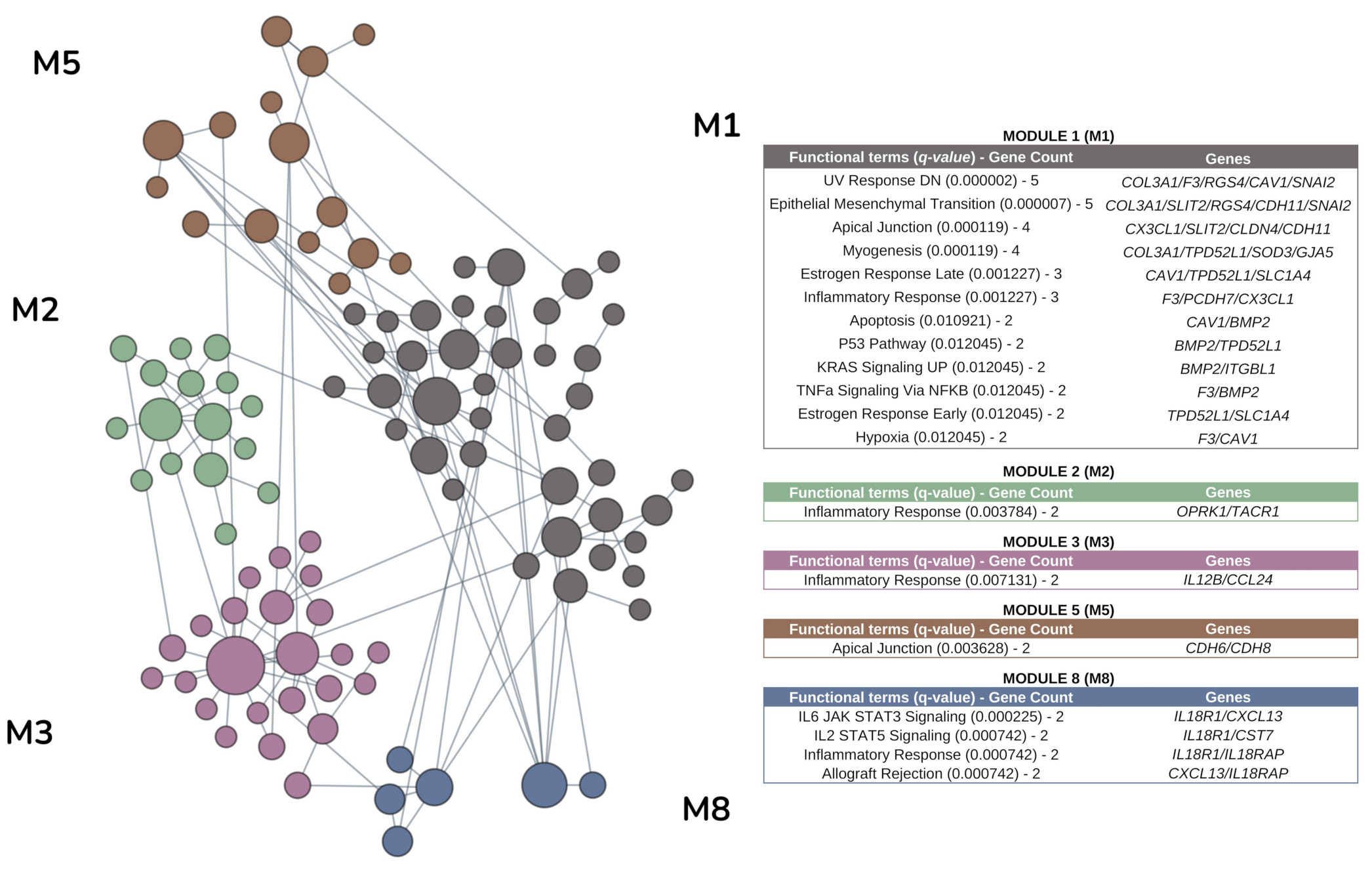

Supplement: Supplementary Figure 5 — Co-expression module from blood tissue-specific network-based functional interpretation of differentially expressed genes paired with DE-HERV. The network represents genes, with the circle size indicating their connection count. The network colors denote the co-expression modules (M). The table details the information corresponding to each module. Generated by the HumanBase tool (https://hb.flatironinstitute.org/) (66). [file Image5.jpg]
